# Acknowledgement of manuscript reviewers 2007–2013

**DOI:** 10.1186/1752-1505-8-1

**Published:** 2014-01-29

**Authors:** Olivier Degomme, Ruwan Ratnayake, Bayard Roberts

**Affiliations:** 1 International Centre for Reproductive Health Ghent University, De Pintelaan 185 UZP114, Ghent 9000, Belgium; 2 International Rescue Committee, 122 E 42nd Street, New York 10168, USA; 3 Faculty of Public Health and Policy London School of Hygiene and Tropical Medicine, 15-17 Tavistock Place, London WC1H 9SH, UK

## Abstract

**Contributing reviewers:**

The editors of Conflict and Health would like to thank all our reviewers who have contributed to the journal from Volume 1 (2007) to Volume 7 (2013).

## 

Antoine Abu-Musa

Lebanon

Hirotsugu Aiga

Japan

Grace Akello

Uganda

Nazmul Alam

United States of America

Samim Al-Dabbagh

Iraq

Joseph Amon

United States of America

Aranka Anema

Canada

Elizabeth Aroka

Kenya

Lynn Muhimbuura Atuyambe

Uganda

Francis Bajunirwe

Uganda

Susan Bartels

United States of America

Linda Bartlett

United States of America

Christian Bautista

Peru

Joseph Becker

United States of America

Marie Theres Benner

Thailand

Theresa Betancourt

United States of America

Joseph Beyene

Canada

Catrien Bijleveld

Netherlands

Oleg Bilukha

United States of America

Karl Blanchet

United Kingdom

Ilse Blignault

Australia

Frederick Burkle

United States of America

German Casas

Colombia

Mustafa Celik

Turkey

Philip Chan

United States of America

Kathryn Chu

South Africa

Alex Cohen

United Kingdom

Manuela Colombini

United Kingdom

Ellen Connorton

United States of America

Aidan Cronin

Ireland

Jeremy Cunningham

United Kingdom

Henia Dakkak

United States of America

Koustuv Dalal

Sweden

Stacy De Jesus

United States of America

Ricardo de la Espriella

Colombia

Sara De Meyer

Belgium

Hedwig Deconinck

France

Hendrik Dierick

Kenya

Shannon Doocy

United States of America

Thomas Elbert

Germany

Tom Ellman

South Africa

Adel Abdel Aziz El-Sayed

Egypt

Karin S Engstrom

Sweden

Ariel Eytan

Switzerland

Nadine Ezard

Australia

Harley Feldbaum

United States of America

Nathan Ford

South Africa

Ralph Frerichs

United States of America

Aleksandra Fucic

Croatia

Richard Garfield

United States of America

Peter Gichangi

Kenya

Nancy Glass

United States of America

Rebecca Grais

France

Natalie Gray

Australia

Gregg Greenough

United States of America

Gilles Guerrier

France

Emily Haroz

United States of America

Paul Harrell

United States of America

Mevludin Hasanović

Bosnia and Herzegovina

Michael Hayes

Canada

Britt Hedman Ahlström

Sweden

Dionisio Jose Herrera Guibert

United States of America

Lara Ho

Switzerland

Jaco Homsy

United States of America

Rebecca Horn

United Kingdom

Mazeda Hossain

United Kingdom

Keng-Yen Huang

United States of America

Peter Hughes

United Kingdom

Robert Hutagalung

Thailand

George Jakab

United States of America

Jamal Jamel

Iraq

Geoffrey Jao

United States of America

Prakash Mohan Jeena

South Africa

Masamine Jimba

Japan

Kirsten Johnson

Canada

Timothy Johnson

United States of America

Rumi Kato Price

United States of America

Jocelyn Kelly

United States of America

Erin Kenny

United States of America

Ines Keygnaert

Belgium

Ali KhajiI

Iran

Brandon Kohrt

Nepal

Ryuichi Komatsu

Switzerland

Sandra Krause

United States of America

Philipp Kuwert

Germany

Chandrakant Lahariya

India

Markus A. Landolt

Switzerland

Erling Larsson

Timor-Leste

Bee Wah Lee

Singapore

Thomas Lee

United States of America

Toby Leslie

United Kingdom

Justin Lessler

United States of America

Shai Linn

Israel

Natalia Linos

United States of America

Augusto Llosa

France

Barbara Lopes Cardozo

United States of America

Francisco López-Muñoz

Spain

Massimo Lowicki-Zucca

Uganda

Kate Mackintosh

Netherlands

Babu Ram Marasini

Nepal

Mendy Marsh

United States of America

Priscilla Martinez

United States of America

Matko Marusic

Croatia

Timothy Mastro

United States of America

Verena Mauch

Germany

Colin McCord

United Kingdom

Therese McGinn

United States of America

Thomas McIntyre

United States of America

Kenneth Menkhaus

United States of America

Steven Miles

United States of America

Edward Mills

Canada

Sharmistha Mishra

Canada

Melinda Moore

United States of America

Neo Morojele

South Africa

Andres Moya

United States of America

Katherine Muldoon

Canada

Luke Mullany

United States of America

Mir Lais Mustafa

Afghanistan

David Ndetei

Kenya

Brett Nelson

United States of America

Richard Neugebauer

United States of America

Marzieh Nojomi

Iran

Colleen O'Connell

Canada

Emilomo Ogbe

Belgium

Peter Olupot-Olupot

Uganda

Donal O'Mathuna

Ireland

Christopher Orach

Uganda

Hermen Ormel

Netherlands

Emilio Ovuga

Uganda

Fritz Parl

United States of America

Bruce Parnell

Australia

Preeti Patel

United Kingdom

Enrico Pavignani

Mozambique

Valerie Percival

Canada

Phuong Pham

United States of America

Jonathan Polonsky

France

Natacha Protopopoff

Tanzania

Andrew Rasmussen

United States of America

Glen Reeves

United States of America

Tony Reid

Belgium

Chen Reis

United States of America

Hugh Reyburn

United Kingdom

Les Roberts

United States of America

Roberto Rona

United Kingdom

Leonard Rubenstein

United States of America

Joseph Rujumba

Uganda

John Russell

United Kingdom

Naomi Rutenberg

United States of America

Tove Ryman

United States of America

Khwaja Mir Islam Saeed

Afghanistan

Alison Schafer

Australia

Gita Sen

India

Nazar Shabila

Iraq

Harry Shannon

Canada

Kate Shannon

Canada

Derrick Silove

Australia

Romesh Silva

United States of America

Sonal Singh

United States of America

Karen Slama

France

Richard Sollom

United States of America

Daya Somasundaram

Sri Lanka

Egbert Sondorp

United Kingdom

Ilene Speizer

United States of America

Paul Spiegel

Switzerland

Hakon Stenmark

Norway

Orly Stern

South Africa

Katharine Stott

South Africa

Alison Strang

United Kingdom

Voravit Suwanvanichkij

United States of America

Raymond Swienton

United States of America

Nathan Taback

Canada

Kate Teela

United States of America

Ara Tekian

United States of America

Andrew Thorne-Lyman

United States of America

John Tirman

United States of America

Wietse Tol

United States of America

Basia Tomczyk

United States of America

Els Torreele

United States of America

Lela Tsiskarishvili

Georgia

Deborah Turnbull

Australia

Marta Valenciano

Spain

Michel Van Herp

Belgium

Mark van Ommeren

Switzerland

An-Sofie Van Parys

Belgium

Beth Vann

United States of America

Joanna Vearey

South Africa

Peter Ventevogel

Netherlands

Patrick Vinck

United States of America

Ulrike von Lersner

Germany

Alexander Vu

United States of America

Angelika Wahner

United States of America

John Watson

Switzerland

Anna Whelan

Australia

Kumanan Wilson

Canada

Andrea Wirtz

United States of America

Viroj Wiwanitkit

Thailand

Bente Wold

Norway

Evan Wood

Canada

Bradley Woodruff

China

Bernadette Wright

Australia

